# Co-production of GroELS discriminates between intrinsic and thermally-induced recombinant protein aggregation during substrate quality control

**DOI:** 10.1186/1475-2859-10-79

**Published:** 2011-10-12

**Authors:** Gemma Platas, Escarlata Rodríguez-Carmona, Elena García-Fruitós, Olivia Cano-Garrido, Antonio Villaverde

**Affiliations:** 1 Institut de Biotecnologia i de Biomedicina, Universitat Autònoma de Barcelona, Bellaterra, Barcelona, Spain; 2 Department of Genetics and Microbiology, Universitat Autònoma de Barcelona, Bellaterra, Barcelona, Spain; 3 CIBER de Bioingeniería, Biomateriales y Nanomedicina (CIBER-BBN), Bellaterra, Barcelona, Spain

## Abstract

**Background:**

The effects and effectiveness of the chaperone pair GroELS on the yield and quality of recombinant polypeptides produced in *Escherichia coli *are matter of controversy, as the reported activities of this complex are not always consistent and eventually indicate undesired side effects. The divergence in the reported data could be due, at least partially, to different experimental conditions in independent research approaches.

**Results:**

We have then selected two structurally different model proteins (namely GFP and *E. coli *β-galactosidase) and two derived aggregation-prone fusions to explore, in a systematic way, the eventual effects of GroELS co-production on yield, solubility and conformational quality. Host cells were cultured at two alternative temperatures below the threshold at which thermal stress is expected to be triggered, to minimize the involvement of independent stress factors.

**Conclusions:**

From the analysis of protein yield, solubility and biological activity of the four model proteins produced alone or along the chaperones, we conclude that GroELS impacts on yield and quality of aggregation-prone proteins with intrinsic determinants but not on thermally induced protein aggregation. No effective modifications of protein solubility have been observed, but significant stabilization of small (encapsulable) substrates and moderate chaperone-induced degradation of larger (excluded) polypeptides. These findings indicate that the activities of this chaperone pair in the context of actively producing recombinant bacteria discriminate between intrinsic and thermally-induced protein aggregation, and that the side effects of GroELS overproduction might be determined by substrate size.

## Introduction

Recombinant protein production is a leading methodological platform of biotechnology and biomedicine [1]. Protein misfolding, degradation, aggregation, inclusion body formation and low functional protein quality are among the main obstacles encountered when using conventional bacterial hosts such as *Escherichia coli *[2-4] as cell factories, these events being specially distressing when proteins are intended for therapeutic uses [5,6]. Apart from shifting to a growing number of alternative hosts, what might eventually improve protein yield and quality, several strategies have been implemented to favour recombinant protein yield and quality in bacteria, specially addressed to enhance proteolytic stability and minimize aggregation [3,7]. As the activities of the cell's quality control were identified ([8], and references therein), the co-production of chaperones along with the target protein was taken as a routine approach. Firstly individual chaperones, and later chaperone-co-chaperone pairs or larger chaperone sets, supplied from additional plasmid vectors, have been assayed as folding modulators under different experimental setting ups and for a large catalogue of proteins [9]. Despite reports of relevant successes, a consensus about final doses and composition of the best chaperone catalogue has not been reached, as the positive effects of this strategy seem to be highly dependent, among other potential factors, on the specific recombinant protein. In addition, externally supplied chaperones might show side effects on the whole physiology of recombinant cells and eventually compromise the quality, solubility or yield of the target recombinant protein [10,11]. Such undesired effects might limit the success of this method, and their occurrence can account, at least partially, for the lack of consistency of chaperone co-production as a generic strategy.

DnaK, a key negative regulator of the quality control system and the main cytosolic *E. coli *chaperone (acting together with its co-chaperone DnaJ) is a common component of the most successful chaperone sets [9,12]. However, an excess of DnaK is recognized to promote functional inactivation of the target protein and eventually its massive proteolysis, at least in given experimental setting ups [13-15]. Protein degradation mediated by DnaK seems to be mechanistically connected to the disaggregation process on the surface of inclusion bodies (where DnaK accumulates), in which by DnaK-DnaJ, IbpA-IbpB and ClpP participate [16]. DnaK-mediated degradation of recombinant polypeptides might represent a physiological replica of σ^32 ^inactivation, through its DnaK-mediated delivery to the protease La during the regulation of the heat shock circuits [17]. Recombinant protein production in DnaK knock out mutants, in contrast, results in high yields of recombinant protein and larger inclusion bodies [18].

The activities of GroEL, the other main cytosolic chaperone in *E. coli*, on recombinant protein solubility, but specially their potential side effects on protein quality are much less known. This might be due to the fact that GroEL knock out mutants are not viable [19], and that studies on partially inactivating *groEL *mutations [20] or truncated versions of the chaperone [21] might render non conclusive results. GroEL co-production has been shown to enhance solubility and/or yield of different proteins, at very different extents (although sometimes with no perceivable or very mild effects) and under different production conditions [22]. GroELS, apart from acting in the sequential folding steps of nascent polypeptides [23-26], is a holding chaperone that can also promote natural and recombinant protein stability and the formation of large macromolecular complexes such as tobacco mosaic virus-like particles [27] and fully assembled P22 phage virions [28], by preventing aggregation and proteolysis of thermolabile intermediates. However, the effects of these chaperones on recombinant protein yield and functional quality are extremely difficult to summarize since diverse authors focus on different analytical parameters, with alternative criteria and under diverse experimental conditions. Also, the potential contribution of thermally induced aggregation of intrinsically aggregation-prone proteins in GroELS activities has been essentially neglected. Beside these findings and considerations, independent observations have again suggested instability of target proteins as potential, undesired effects associated to GroEL overproduction [10], in special, proteolysis of proteins deposited as inclusion bodies [29].

## Results

To examine in a defined experimental setting up, how GroEL and its co-chaperone GroES can affect the yield, stability and quality of aggregation-prone polypeptides, and to identify mechanistic roles of this chaperone pair in the quality control of recombinant proteins we have selected as models two structurally dissimilar soluble proteins (namely GFP and *E. coli *β-galactosidase). These polypeptides were produced as non-fused, pseudo wild type versions or joined to a hydrophobic viral protein (the VP1 capsid protein of foot-and-mouth disease virus) that acts as an efficient aggregation tag. While GFP is a monomeric small protein (27 kDa) that gains activity through a maturation process in the last folding steps [30], β-galactosidase is a huge tetrameric protein (470 kDa) that becomes active once the correct contacts between the monomer's interfaces have been stabilized [31]. Point mutations in GFP have a dramatic impact on the aggregation rate of the protein [32] while disposition of both GFP (and related fluorescent proteins) and β-galactosidase as inclusion bodies is also sensitive to the amino acid sequence and position of end-terminal fused aggregation tags [33-35]. Aggregation-prone versions of both proteins have been shown to interact in vivo with GroEL [36-38].

**Figure 1:**
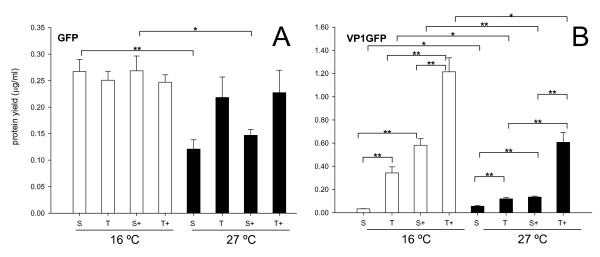
**Influence of GroELS co-production on yield and solubility of native and engineered GFPs**. Yield of total (T) and soluble (S) GFP **(A) **and VP1GFP **(B) **produced alone or along with the chaperones GroELS (+). Only significant differences between relevant data pairs are indicated as *, 0.01 <*p *< 0.05 and ** *p *< 0.01.

To minimize the contribution of thermal stress on protein deposition we tested recombinant protein production at two suboptimal growth temperatures, namely 16°C and 27°C. The non-fused GFP was fully soluble when produced in *E. coli *at 16°C (Figure 1A). However, around 40% of recombinant GFP occurred in the insoluble cell fraction when the culture temperature was up-set to 27°C (Figure 1A). Co-production of GroELS did not improve GFP solubility in any of these production conditions (Figure 1A). Also, GFP yield was very similar in all cases, excluding the possibility of extended proteolysis mediated by the chaperones. In contrast, the aggregation-prone version of the same protein (VP1GFP) was mainly insoluble at both 16°C and 27°C (Figure 1B), indicating an intrinsic rather than environmental-induced trend to deposition mediated by the viral tag. The yield of VP1GFP at 27°C was significantly lower than that at 16°C, suggesting proteolysis of this protein. However, and in contrast with GFP, the co-production of GroELS dramatically enhanced the yield of VP1GFP (Figure 1B). Furthermore, VP1GFP solubility, expressed as the ratio of protein occurring in the soluble fraction over total protein, was not dramatically enhanced by the chaperones at 27°C (Figure 1B). The five-fold increase in solubility observed at 16°C might be a numerical rather than a factual issue linked to data scattering at low yield values.

**Figure 2:**
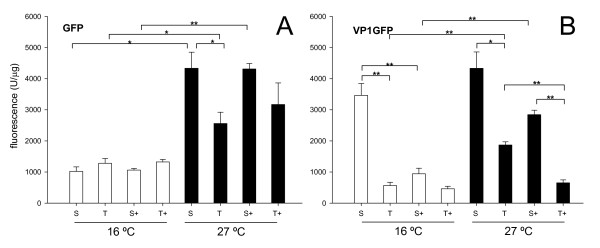
**Influence of GroELS co-production on the conformational quality of native and engineered GFPs**. Specific fluorescence emission of total (T) and soluble (S) fractions of GFP **(A) **and VP1GFP **(B) **alone or along with the chaperones GroELS (+). Specific fluorescence was calculated as the ratio of fluorescence versus recombinant protein amounts. Only significant differences between relevant data pairs are indicated as *, 0.01 <*p *< 0.05 and ** *p *< 0.01.

When exploring GFP conformational and functional quality through the fluorescence emitted per mass ratio, higher conformational quality was immediately evidenced at 27°C (Figure 2A), indicative of a more efficient protein maturation. At this temperature, the soluble protein version was slightly more fluorescent than the aggregated counterparts, as expected. GroELS had no detectable effect on the fluorescence emission of GFP at any temperature (Figure 2A). A similar global profile was observed during the production of VP1GFP. However, in this case, the chaperone complex minimized the fluorescence emission of both soluble and aggregated versions of VP1GFP (Figure 2B), whose yield was enhanced by the chaperones.

**Figure 3:**
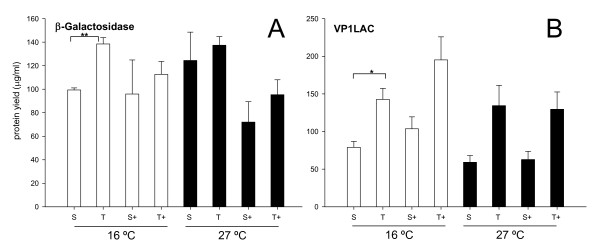
**Influence of GroELS co-production on yield and solubility of native and engineered β-galactosidases**. Yield of total (T) and soluble (S) β-galactosidases **(A) **and VP1LAC **(B) **produced alone or along with the chaperones GroELS (+). Only significant differences between relevant data pairs are indicated as *, 0.01 <*p *< 0.05 and ** *p *< 0.01.

**Figure 4:**
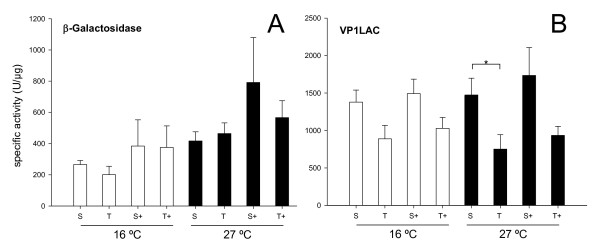
**Influence of GroELS co-production on the conformational quality of native and engineered β-galactosidases**. Specific activity of total (T) and soluble (S) β-galactosidases **(A) **and VP1LAC **(B) **produced alone or along with the chaperones GroELS (+). Only significant differences between relevant data pairs are indicated as *, 0.01 <*p *< 0.05 and ** *p *< 0.01.

β-Galactosidase was, as in the case of GFP, essentially soluble and both protein yield and solubility essentially unaffected by the external supply of GroELS (Figure 3A). On the other hand, 50% of recombinant VP1LAC was found in the insoluble cell fraction irrespective of GroELS co-production (Figure 3B). No temperature-induced aggregation was observed in any case. Again, no symptoms of proteolysis were manifest regarding any of the β-galactosidase versions (Figure 3), although a minor, non significant reduction of β-galactosidase yield observed at both 16°C and 27°C could be compatible with GroELS-mediated degradation (Figure 3A). In this line, and irrespective of the events supporting the lower yield, the enzymatic activity of non-fused β-galactosidase was concomitantly higher in presence of externally supplied GroELS (Figure 4A). Again, this trend was not statistically significant although the inverse coincidence between yield and specific activity of the protein was evident. In contrast, the yield and functional quality of the largest protein version VP1LAC were essentially regular and it was unaffected by the chaperone pair (Figures 3B and 4B).

In conclusion, co-production of GroELS along with non-fused, full length GFP did not have any effect on protein yield, stability and quality at 16°C (Figure 1A), conditions under which this protein was fully soluble. At 27°C, GFP partially aggregated, indicating thermal aggression and conformational effects even at this mild temperature. GroELS pair was not able to recover the solubility of the protein under these conditions (Figure 1A), and neither its functional quality expressed as specific fluorescence emission (Figure 2B). On the other hand, no signs of GFP or VP1GFP proteolysis associated to GroELS production were observed (Figure 1), although a slight reduction in the yield of β-galactosidase was evidenced in presence of the recombinant chaperones (Figure 3), that might be compatible with the occurrence of degradation. Contrarily, GroELS dramatically enhanced the yield of VP1GFP (Figure 2), what could be in turn associated to a stabilizing effect of the chaperone pair on the chimerical protein. Concomitantly, GroELS reduced the fluorescence emission of both soluble and insoluble VP1GFP versions (Figure 2B).

## Discussion

The examination of the solubility and conformational quality of non-fused and chimerical versions of two structurally different model proteins indicate that the GroELS chaperone pair does not modulate the folding of thermally-injured GFP, but instead that of the aggregation-prone version of this protein (VP1GFP), which size (around 50 kDa) should still permit its encapsulation by GroELS complexes [39]. This suggests a differential role of GroELS in thermal induced and intrinsic aggregation pathways of their substrates. Although there is not straightforward experimental data fully demonstrating this hypothesis, data presented in Figure 1B suggest a proteolytic stabilization of VP1GFP that enhances its yield. In agreement with previous observations [15], such an increase in the intracellular concentration of the chimerical protein reduces its conformational quality, in both soluble and insoluble cell fractions (Figure 2). This fact indicates that recombinant protein yield and quality are divergent features and supports the concept that both parameters cannot be simultaneously enhanced in *E. coli *[40], at least for aggregation-prone proteins. Such a divergence is also supported by the slight increase of β-galactosidase activity mediated by GroELS concomitant to the reduction of protein yield (Figures 3A and 4A). Also, the parallel behaviour regarding functional activity of soluble and insoluble GFP versions is in the line of the "continuum-of-forms" model of recombinant protein production in bacteria [41], that claim for the extreme dynamism of protein aggregation-disaggregation [37] and that offers a mechanistic explanation about why insoluble protein species are not excluded from the cell's quality control [42]. GroELS was not able to positively reduce aggregation of VP1GFP (Figure 1B), a role that seems limited to DnaK and associated AAA^+ ^disaggregating proteins [43].

Finally, overproduction of GroELS shows extremely mild effects on the production of two versions of the large β-galactosidase protein, which cannot be encapsulated by GroEL rings. This is in agreement with previous observations of GroEL null effects on the folding of RNA polymerases [44] and other large proteins [10]. In our hands, GroEL tends to reduce the yield of β-galactosidase without affecting the partitioning between soluble and insoluble cell fractions, suggesting a negative role of this chaperone on protein stability, as shown before for a limited set of abnormal and native cellular proteins [45-48]. In this regard, it has been observed that GroEL interacts externally (without the cooperation of GroES) with proteins too large to be encapsulated [49]. The negative influence of GroELS on protein yield, although no statistically significant, is supported by the rise of the specific enzymatic activity in GroELS-producing cells.

In summary, we have not observed any positive effect on the yield, production and folding of thermally aggregated GFP but positive GroELS activities on the yield of an aggregation-prone version of GFP (VP1GFP). Variations in the nominal solubility of these proteins (determined as soluble protein amounts over total protein) during overproduction of the chaperone pair seem to be irrelevant numerical alterations of these ratios derived from data scattering, as no solubilising effects of GroELS were in any case evidenced through a reduction of the absolute amounts of insoluble species. The increase of VP1GFP yield, especially dramatic at 16°C, can be attributed to protein stabilization. Contrarily, GroELS co-production has mild (if any) effects on large proteins that cannot be encapsulated, on which, however, the chaperone pair tends to reduce their proteolytic stability.

## Methods

### Bacterial strain, plasmids and proteins

The *E. coli *strain (MC4100 (*araD139 (argF-lac)U169 rpsL150relA1 flbB5301 deoC1 ptsF25 rbsR*)) was used in all the experiments. The recombinant proteins were produced by expressing the encoding genes from the ampicillin-resistant pTrc99A-derivatives pTGFP (encoding GFP), pTVP1GFP (encoding VP1GFP), pTCO46 (encoding a pseudo-wild type β-galactosidase) and pTVP1LAC (encoding VP1LAC). No leader peptides were fused in any case so all the proteins remained in the cell's cytoplasm. More details about these constructs where provided elsewhere [15,33,35]. GroELS pair was supplied from the plasmid pBB541 (Km^R^), kindly provided by Prof B. Bukau.

### Culture and sampling conditions

LB medium [50] with the corresponding plasmid-maintenance antibiotics (ampicilin at 100 μg/ml and kanamycin at 60 μg/ml, when necessary) was used in all the experiments. Cells from overnight cultures were diluted at 1:50 in 120 ml of fresh media and grown at either 16°C or 27°C, within 500 ml shake flasks, at 250 rpm. Induction of gene expression (encoding both the models protein and the chaperone set) was triggered by the addition of 1 mM IPTG at an OD_550 _= 0.5. Cultures were always done in triplicate to ensure statistic robustness.

### Sampling and protein analysis

Samples of 15 ml were taken when OD_550 _reached 2-3, after the induction of gene expression and cells sedimented by low speed centrifugation at 6.000 g for 10 min. Cells were disrupted by sonication as described [51] and soluble cell fraction recovered by centrifugation at 15.000 g for 15 min. Total cell extracts and the separated soluble cell fraction were run in PAGE-electrophoresis as described [15] and protein bands identified by further Western blot analysis, using commercial anti-GFP and anti-β-galactosidase antibodies as described [35]. The amount of proteins was determined by Quantity One^® ^software using dilutions of commercial GFP and β-galactosidase proteins as standards. Fluorescence emission and β-galactosidase enzymatic activity were determined by standard fluorimetry or enzymatic determination (Miller's method) respectively [35]. Significance of differences between relevant data pairs were evaluated by a Student's t-test.

## Competing interests

The authors declare that they have no competing interests.

## Authors' contributions

GP, ERC, EGF and OC performed the experiments, processed data and prepared the graphic files. ERC, EGF and AV conceived and designed the experimental approach. AV coordinated the whole study and redacted and prepared the manuscript in its final form. GP, ERC, EGF have equally contributed to this work. All authors read and approved the final manuscript.
